# Stem Cells Associated with Adult Skeletal Muscle Can Form Beating Cardiac Tissue In Vitro in Response to Media Containing Heparin, Dexamethasone, Growth Factors and Hydrogen Peroxide

**DOI:** 10.3390/ijms26062683

**Published:** 2025-03-17

**Authors:** Leonard M. Eisenberg, Carol A. Eisenberg

**Affiliations:** Department of Physiology, New York Medical College, Valhalla, NY 10595, USA; leonard_eisenberg@nymc.edu

**Keywords:** heart, cardiac myocyte, differentiation, cell culture, contractility, skeletal muscle, dexamethasone

## Abstract

Both cardiac and skeletal muscles originate from the mesoderm, although the two tissues develop from distinct primordia within the early embryo. The shared, albeit distinctive muscle phenotype of these two cell types have led many researchers to investigate whether stem cells from adult skeletal muscle have the capacity to generate cells with a contractile, cardiac phenotype. To date, most of those studies have relied on multistep protocols requiring tissue engineering, co-cultures or transplantation experimentation. In this report, we describe a simple, cell culture method for obtaining contractile, cardiogenic aggregates from skeletal muscle-derived stem cells (MDSCs). Combining in vitro conditions used for promoting the differentiation of cardiac progenitor cells and the long-term maintenance of heart tissue fragments, we have been able to convert MDSCs to myocardial cells that aggregate into beating myospheres. These selective and optimized culture conditions continued to support a contractile cardiogenic phenotype for over four months in vitro. This culture protocol provides a model for future insights into the pathways responsible for the divergence of skeletal and cardiac phenotypes, as well as a source of easily obtained myocardial tissue for subsequent scientific investigations into cardiac function and biology.

## 1. Introduction

Cardiac and skeletal myocytes arise from distinct progenitor fields within the mesoderm layer of the early embryo. Cardiac myocyte progenitors first appear soon after gastrulation begins, in the precardiac mesoderm (also referred to as the heart-forming fields and primary heart fields), which is distributed bilaterally to the anterior portion of the primitive streak [1,2]. As the precardiac mesoderm begins to develop into the primary heart tube, the adjoining secondary heart field begins to contribute cardiac myocyte progenitors to the newly forming heart [3,4]. Shortly after the specification of the primary heart field, skeletal myocyte progenitors begin to appear within the somites, which are the transient metameric segments that appear along both sides of the developing neural tube [5,6]. Because of the different locations of the cardiac and skeletal myocyte progenitor/stem cells within the embryo, and later in the adult, these two muscle cell types have long been considered to arise from distinct cell lineages—that is, the two types of striated muscle are thought to arise from dissimilar stem cell populations. Support for the complete distinctiveness in these lineages was also based on the functional differences exhibited by these different varieties of striated muscle, the distinct myofibril isoforms that are exhibited by the two muscle types and the various differences in the molecular makeup of these cells that account for their contrasting functional properties [7,8,9]. Moreover, the differentiation of skeletal and cardiac muscles is driven by transcriptional protein pathways that are unique to each cell type [10,11,12].

Yet, during the earliest stages of differentiation, cardiac and skeletal myocytes exhibit many overlapping properties. There is the transient expression of smooth muscle markers during the initial stages of both cardiac and skeletal myocyte development [13,14,15]. Even later, when cells start to first exhibit definitive cardiac and skeletal muscle phenotypes, the two striated muscle types will exhibit similar myofibrillar isoform profiles. For example, during development, skeletal and cardiac muscles co-express both skeletal and cardiac α-actin genes [16]. Both the developing heart and skeletal muscle express the adult skeletal muscle-associated MYH4 myosin heavy-chain (MyHC) isoform, and cardiac troponin I (cTn-I) [17]. Likewise, the MYH7 gene that encodes the β-myosin heavy-chain isoform is expressed in cardiac and type I skeletal muscle fibers [18,19]. Even the well-known skeletal muscle transcription factor, MyoD, has been detected in the embryonic heart [20,21]. Together, these data suggest that developing cardiac and skeletal muscle cell types may not necessarily arise from distinct mesodermal lineages.

These transient similarities in the two muscle types, at least in the earliest stages of differentiation, have led many scientists to explore whether the relatively accessible skeletal muscle stem cells from adult tissues might serve as a cellular source for generating new cardiac muscle [22,23]. To date, the results have been ambiguous, as skeletal stem cells provoked to undergo cardiac differentiation have often generated either mixed cultures of cardiac and skeletal muscles, cells that only underwent partial cardiac differentiation, and/or hybrid cardiac and skeletal myocyte phenotypes [24,25,26,27,28].

In the present study, we investigated whether skeletal-muscle-derived stem cells were able to generate contractile cardiac tissue that was free from contaminating skeletal muscle cells. As a starting point for our investigations, we applied conditions that we had used previously for generating cardiac tissue from cardiac progenitors obtained from the early embryo and adult heart. In addition, we employed in vitro protocols that helped coax partial cardiac differentiation from bone marrow stem cells, as well as protocols we established for maintaining the long-term maintenance of a contractile phenotype from adult cardiac tissue explants [29,30,31,32].

To further examine the mesoderm lineage pathway shared by both cardiac and skeletal muscles, we explored the differentiation capacity of stem cells found within adult skeletal muscle. The objectives of these studies were to develop in vitro culture conditions that would select and promote a cardiomyocyte phenotype from tissue containing cells normally fated to produce skeletal muscle. As described below, our experimentation yielded conditions that selectively promote contracting cells expressing proteins associated with cardiomyocytes. Furthermore, the development of a skeletal muscle phenotype was not detected, with skeletal muscle markers being absent within the cultures. Most importantly, these cultures conditions continued to support a contractile cell phenotype for greater than four months in vitro.

## 2. Results

### 2.1. Characterization of Skeletal Muscle Stem Cells

**Figure 1:**
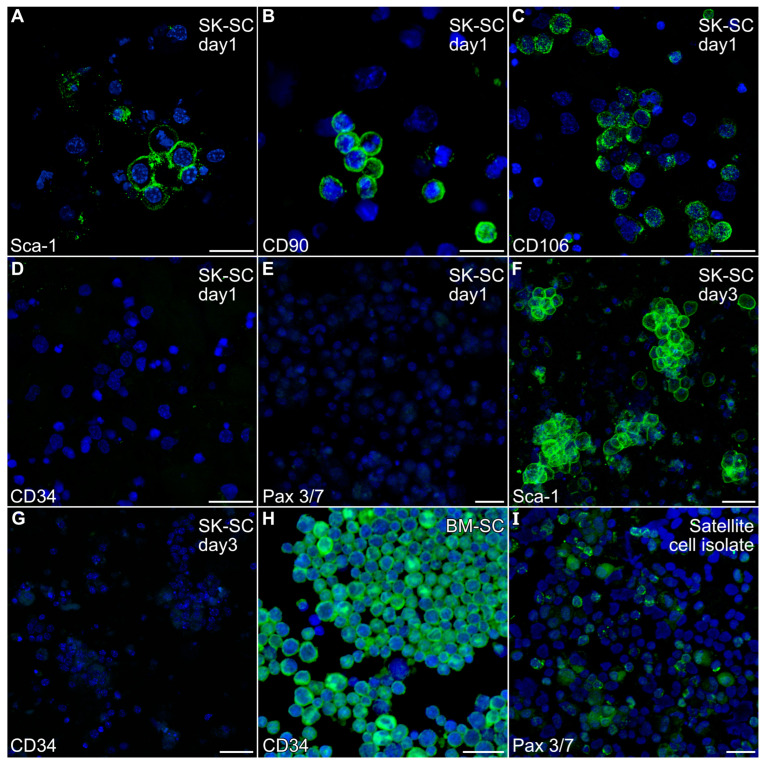
Expression of stem cell markers by non-adherent hindlimb muscle-derived cells. The non-adherent cell population harvested from mouse hindlimb muscle was (**A**–**E**) cytospun after the first day of culture or (**F**,**G**) directly fixed in the dishes at day 3, before being fluorescently stained with antibodies specific for (**A**,**F**) Sca-1, (**B**) CD90, (**C**) CD106, (**D**,**G**) CD34 or (**E**) Pax 3/7 antibodies (green), followed by counterstaining with the nuclear DAPI label (blue). The immunofluorescent labeling of the cells indicated that the muscle-derived cells were Sca-1-, CD90- and CD106-positive, but CD34- and Pax 3/7-negative. (**H**) Positive control bone marrow stem cells and (**I**) satellite cell isolates were stained in parallel to verify the immunoreactivity of the CD34 and Pax 3/7 antibodies, respectively. Scale bar = 25 µm.

A non-adherent cell population was harvested from mouse hindlimb muscle by enzymatic treatment of the finely minced muscle tissue, followed by repetitive cell dissociation with a 20-gauge needle. After filtering and centrifugation, the resulting cell population obtained from the mouse hindlimb was cultured overnight in low serum to remove adherent cells prior to immunocytochemical analysis. Non-adherent cells were harvested and fluorescently stained with antibodies specific for Sca-1, CD34, CD90, CD106, NG2 and Pax3/7 antibodies, which are markers ascribed to various stem cell populations associated with skeletal muscle [23,33,34,35]. As is shown in Figure 1A–C, the non-adherent cells obtained from skeletal-musclewere Sca-1-, CD90- and CD106-positive. In contrast, these cells were negative for CD34 (Figure 1D), Pax3/7 (Figure 1E) and NG2

Our previous experiments with mouse bone marrow demonstrated that the growth of adult stem cells was well supported by incubating with IMDM with 20% FBS [32,36]. Similar culture conditions have been used to promote skeletal muscle differentiation from hindlimb tissue preparations [37]. Thus, we expanded stem cell populations obtained from adult murine hindlimb muscles by short-term culturing of the muscle-derived stem cells (MDSCs) in IMDM with 5% FBS, EGF and bFGF. Within 3 days, the non-adherent cells began to cluster, which we retested for expression of the stem cell markers. These cells still retained Sca-1 expression (Figure 1F) and remained CD34-negative (Figure 1G). In contrast, positive control bone marrow stem cells and stem cell isolates stained intensely for CD34 and Pax3/7 expression, respectively (Figure 1H,I).

**Figure 2:**
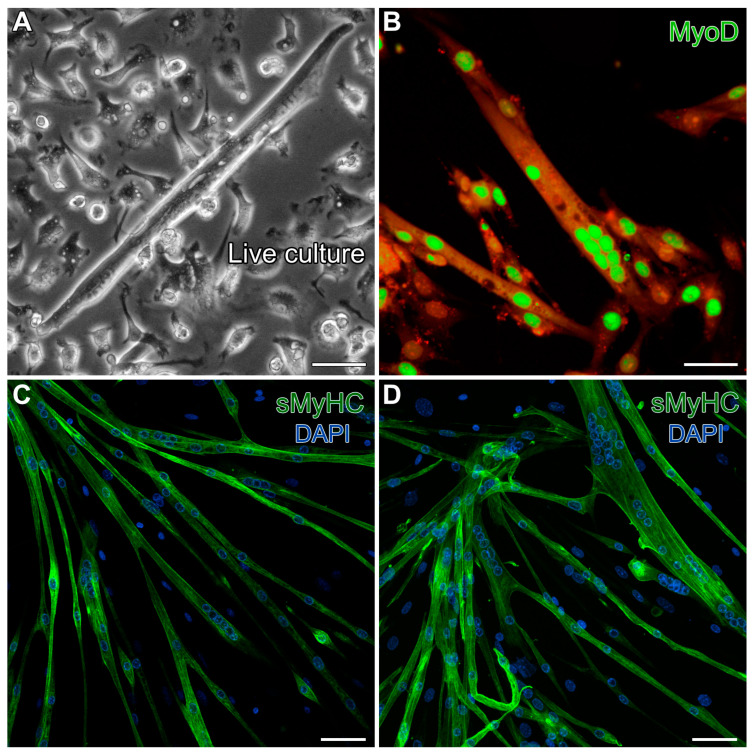
Verification of the skeletal muscle potential of MDSCs obtained from the mouse hindlimb. Day-13 MDSC cultures grown under standard skeletal myogenic differentiating conditions exhibited cells (**A**) displaying the typical skeletal myotube morphology in live cultures. (**B**) These multinucleated cells exhibited prominent immunolabeling for the skeletal-muscle-specific transcription factor MyoD (green). For this image, high gain was used to bring out autofluorescence in the red channel to show the contours of the myotubes. (**C**,**D**) Fully differentiated skeletal myotubes were verified by fluorescent labeling for sMyHC (green), as shown in conjunction with the nuclear DAPI counterstain (blue). Scale bar = 20 µm.

As a final characterization of the MDSCs we obtained from the mouse hindlimbs, we tested for the ability of these cells to differentiate under standard conditions for generating skeletal muscle. Following one week of cell growth with 20% FBS, the serum levels were reduced to 5%, but with added insulin, to promote skeletal muscle development. These conditions promoted 40–50% confluent cultures by day 5, and by day 13, the cultures produced cells exhibiting the typical skeletal myotube morphology (Figure 2A). These multinucleated cells displayed prominent staining for the skeletal-muscle-specific transcription factor MyoD (Figure 2B) and exhibited sarcomeric protein expression throughout the myotubes, as indicated by fluorescent labeling for sarcomeric myosin heavy-chain (sMyHC) (Figure 2C,D). These results verified that our isolation protocol yielded progenitor/stem cells displaying the expected skeletal muscle phenotypic potential.

### 2.2. Aggregation of Skeletal Muscle Stem Cells and Cardiac Differentiation

**Figure 3:**
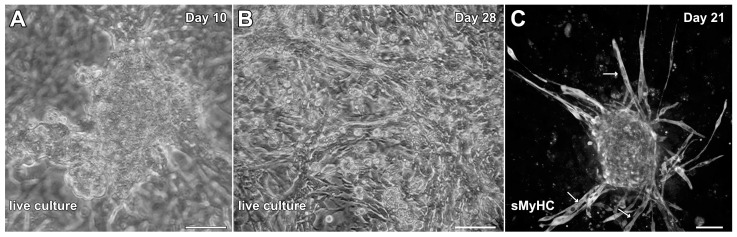
Initial experimentation promoting cardiac differentiation from aggregated MDSCs. MDSCs were exposed to media conditions that were used previously to promote cardiogenesis of adult mouse CPCs and cardiac tissue explants. In response to incubation in media containing EGF, bFGF, H_2_O_2_ and dexamethasone, MDSCs began to cluster into aggregates and give rise to contractile tissue as early as day 8 of culture. (**A**) Beating cardiac myosphere as imaged on day 10. Over time, the frequency of contractility increased. (**B**) However, as shown at day 28, beating myospheres flattened and spread out onto the dish, although the tissue remained contractile. (**C**) Immunostaining cultures at day 21 for sMyHC revealed the appearance of long spindle-shaped cells (arrows) within the dishes, which indicated that contaminating skeletal muscle cells were developing along with beating cardiomyocytes. Scale bar = 50 µm.

To determine whether cells associated with hindlimb skeletal muscle had cardiac potential, stem-cell-enriched cell suspensions obtained from this tissue were exposed to factors that we had used previously to promote cardiac differentiation from adult mouse cardiac progenitor cells (CPCs) and maintain the cardiac phenotype of cardiac tissue explants in the long term [30,31]. These factors included EGF, bFGF, H_2_O_2_ and dexamethasone. The non-adherent cells obtained from the skeletal muscle tissue began to attach to the culture substrate and cluster into aggregates by day 3, which started to display a muscle phenotype as early as day 6. Contractile activity by the aggregates was first observed by day 8 and became more pronounced by day 10 (Figure 3A; Video S1). By 2 weeks of culture, beating cultures were more frequently observed. However, myocyte cultures at 4 weeks of incubation, although highly contractile (Video S2) were often accompanied by the flattening and spreading out of the previously aggregated tissue within the dishes (Figure 3B). More unwanted was that long spindle-shaped cells began to appear within the cultures after two weeks of incubation (Figure 3B,C), which indicated the presence of skeletal muscle cells mixed with the beating cardiac tissue. Thus, we decided to ascertain whether we could improve the culture protocol to generate cardiac tissue from hindlimb muscle without the contaminating skeletal muscle cells.

### 2.3. Formation of Contractile Cardiac Tissue Without Skeletal Muscle Contamination

**Figure 4:**
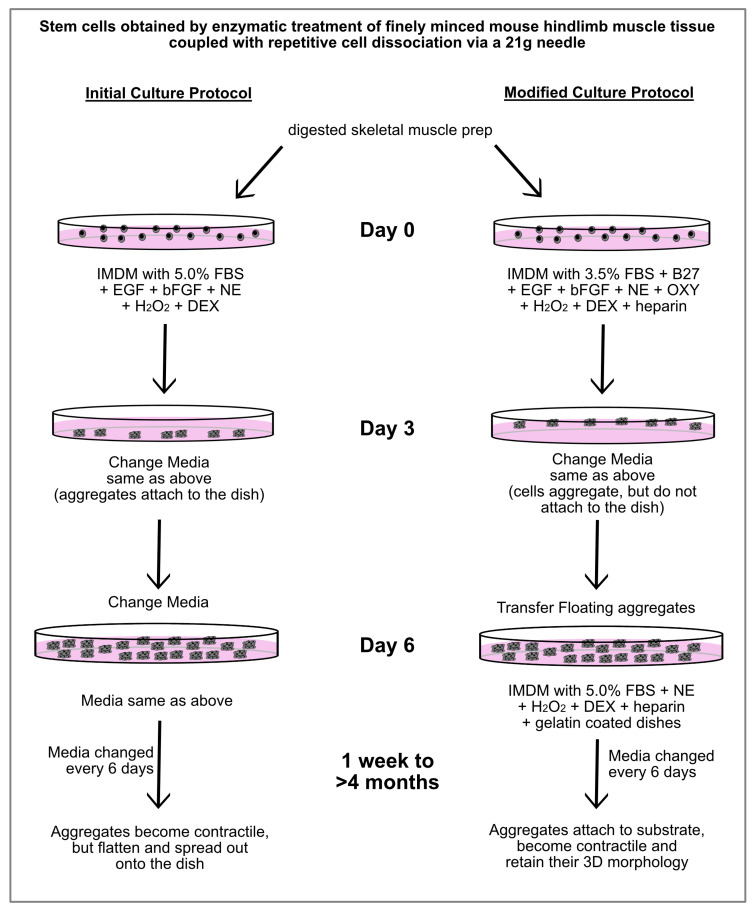
Outline of culture protocols used to obtain contractile cardiac tissue from MDSCs. Schematic diagram showing the initial and modified culture protocols for generating contractile cardiac myospheres from MDSCs. Under the initial culture protocol that we formulated, beating myospheres were obtained. However, over time the aggregates flattened and spread out onto the dish, with contaminating skeletal muscle cells being observed. In contrast, MDSCs cultured with the modified protocol produced beating cardiac myospheres that maintained their 3D morphology for >4 months. Under these modified conditions, neither cells exhibiting a typical skeletal morphology nor MyoD-positive expression were observed. Abbreviations used exclusively in this diagram: NE (norepinephrine), OXY (oxytocin) and DEX (dexamethasone).

After much trial-and-error experimentation, we were able to develop a protocol that produced contractile cardiac tissue from MDSCs, without supporting skeletal muscle differentiation. As is outlined in Figure 4, the first alternations in the protocol were made for the first 6 days of incubation, which prevented the aggregates that formed from the hindlimb-muscle-derived stem cells from attaching to the culture dishes. Four changes in the stem cell media were incorporated in the modified protocol for the first 6 days of culture: (1) reducing the FBS concentrations from 5.0% to 3.5%; adding (2) B27 supplement to compensate the cells for the reduced FBS; (3) oxytocin, which is a characterized cardiomorphogen that promotes cardiac differentiation [38,39]; and (4) heparin. The purpose of the heparin was to keep the 3D shape of the aggregates and prevent the tissue from flattening out—which our previous studies have shown is an essential attribute for supporting the long-term maintenance of a contractile cardiac phenotype [29,30]. The resulting aggregates were then collected after the first 6 days of incubation and transferred to new dishes which were now gelatin-coated. The media used for this second phase was similar to that just described, except the FBS concentration was increased back to 5.0%, and without EGF, bFGF, B27 and oxytocin.

**Figure 5:**
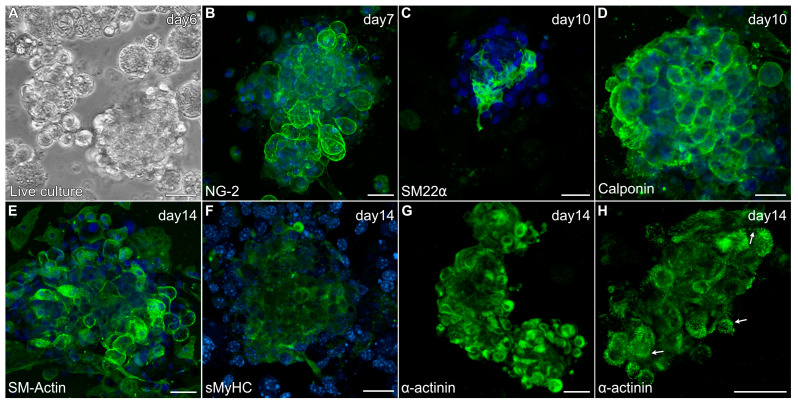
Characterization of MDSC-derived myospheres during the first two weeks of incubation under the modified culture protocol. (**A**) Floating aggregate culture imaged after 6 days of incubation. (**B**–**H**) Aggregate cultures were immunolabeled for various proteins (green) and counterstained with the nuclear marker DAPI (blue). Within days of being transferred to new dishes, aggregated tissue exhibited expression for (**B**) NG2, (**C**) SM22α and (**D**) calponin. Myospheres imaged at two weeks showed (**E**) prominent SM-actin expression, (**F**) sMyHC and (**G**) α-actinin staining at the periphery of cells within the aggregates. (**H**) Higher magnification view of α-actinin shows a striated pattern at the cell periphery (arrows), which is reminiscent of the pattern of sarcomeric organization in cultures of early embryonic cardiac tissue. Scale bar = 25 μm.

In response to the modified culture protocol, non-adherent cells obtained from the skeletal muscle tissue began to form small aggregates by day 3 (Figure 5A). After 6 days of culture, floating aggregates were transferred to new dishes, where they would begin to loosely attach to the culture surface in response to the gelatin coating. These early cultures (days 6–8) demonstrated positive staining for several proteins associated with newly differentiated cardiomyocytes that are shared by smooth muscle cells, including NG-2 (Figure 5B), SM22α (Figure 5C), calponin (Figure 5D) and smooth muscle (SM)-actin (Figure 5E). Additionally, the aggregates began exhibiting sMyHC (Figure 5F) and α-actinin (Figure 5G) expression—with the latter proteins showing the formation of sarcomeric pattering at the cell periphery (Figure 5H), which is very characteristic of cardiac tissue formation from precardiac cells obtained within the early embryo [29,40].

**Figure 6:**
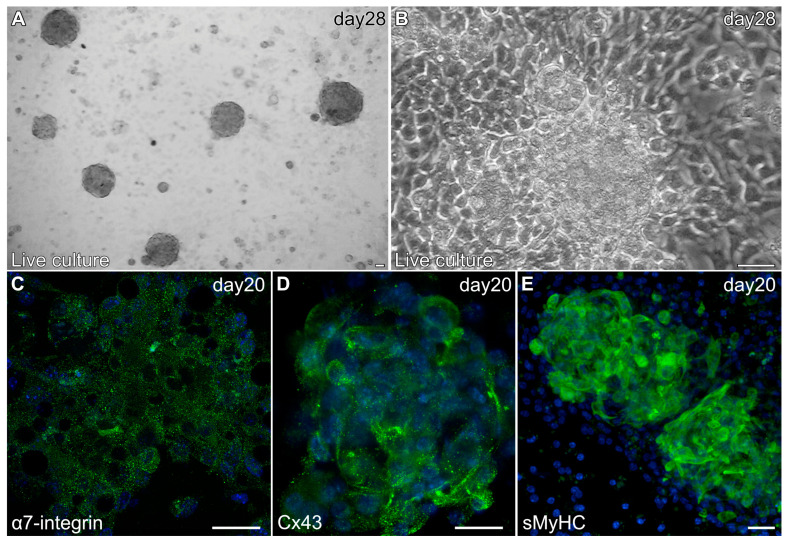
Characterization of MDSC-derived myospheres during the second two weeks of incubation under the modified culture protocol. (**A**) Brightfield image of a 28-day culture at low magnification showing the presence of multiple contractile myospheres that have retained their 3D morphology. (**B**) Live culture of an individual 28-day contractile myosphere imaged in phase. Note that the cells neighboring the contractile myosphere are cuboidal, and that there is an absence of cells surrounding the aggregated tissue that exhibited an elongated skeletal myocyte cell shape. Myospheres imaged at day 20 displayed expression (green) of (**C**) α7-integrin, (**D**) Cx43 and (**E**) sMyHC; along with DAPI nuclear counterstaining (blue). Scale bar = 25 μm.

**Figure 7:**
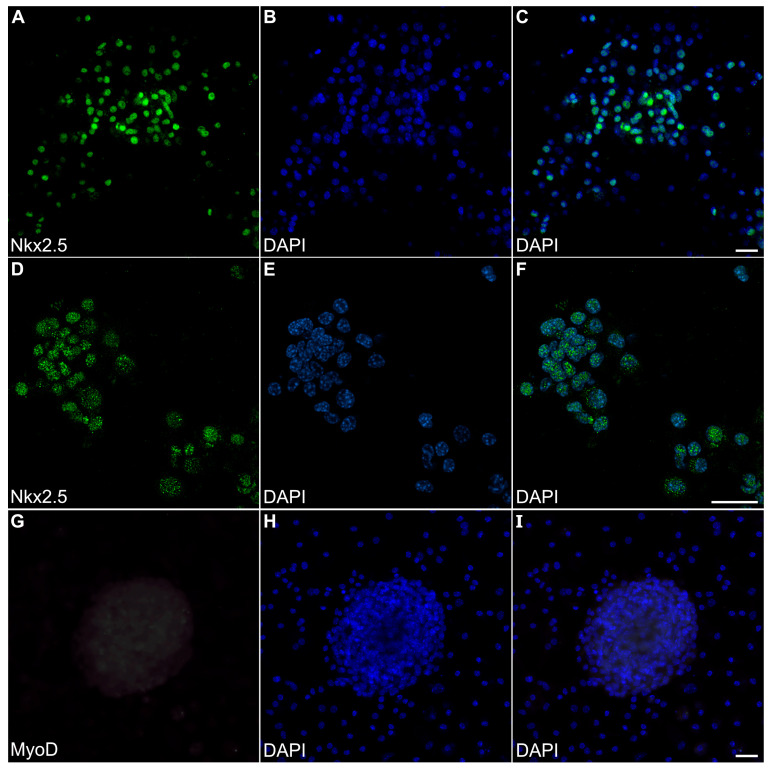
Transcription factor expression in myosphere cultures. MDSC-derived myosphere cultures at day 20 were imaged for fluorescently labeled antibodies (green) and DAPI counterstained (blue) to detect nuclei, as shown individually (left and middle columns, respectively) and as a merged image (right columns. (**A**–**C**) Myosphere stained for the cardiac-myocyte-associated transcription Nkx2.5, along with the corresponding nuclear labeling with DAPI. (**D**–**F**) A second myosphere that developed from a different experimental batch is shown at a higher magnification for both Nkx2.5 immunostaining and DAPI fluorescence. Note that these myospheres exhibit NKx2.5 immunoreactivity that localize to the nuclei. (**G**) In contrast, day-20 myosphere cultures did not exhibit immunostainingfor the skeletal-muscle-associated MyoD transcription factor, including within the nuclei that are indicated by (**H**,**I**) DAPI labeling. Compare the staining of the bottom panels to the positive MyoD staining for myotubes imaged in Figure 2. Scale bar = 25 μm.

**Figure 8:**
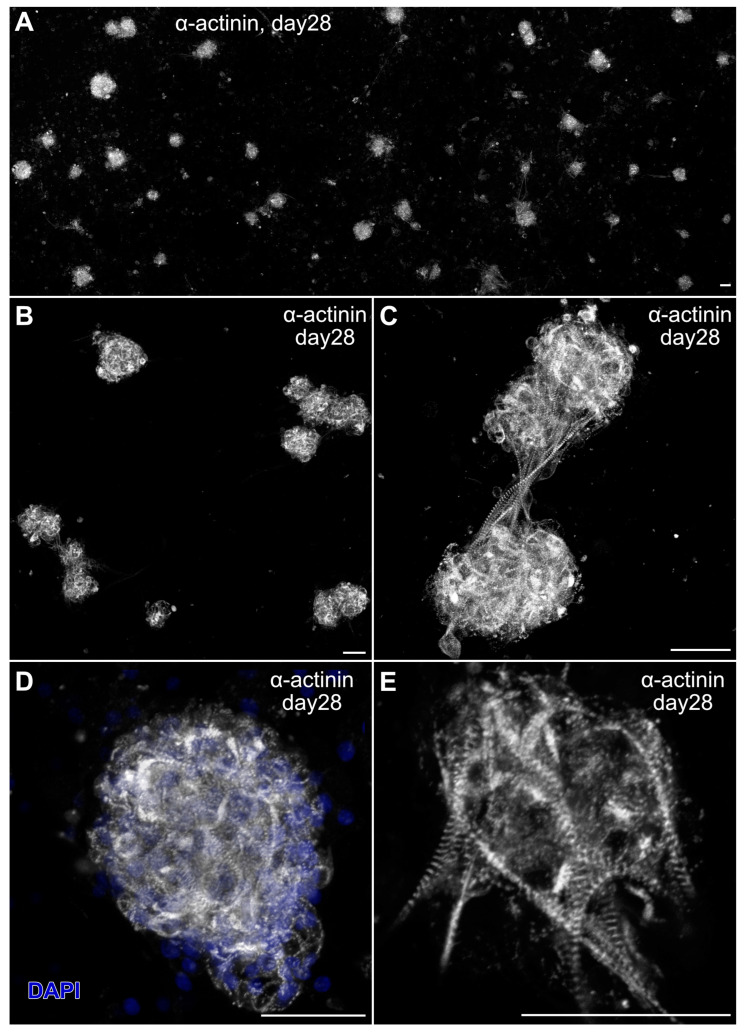
Sarcomeric organization in myosphere cultures. MDSC-derived contractile myosphere cultures at day 28 were fluorescently labeled with α-actinin (gray). (**A**) Panoramic view of the cultures at low magnification that show the distribution of cardiac myospheres present in these cultures. (**B**–**E**) Individual fields were imaged at successively higher magnification, with panel (**D**) also displaying the DAPI nuclear counterstain (blue). Note the striated pattern of α-actinin immunolabeling, which is indicative of the developed myofibrillar organization of the beating MDSC-derived cardiac tissue. Scale bar = 50 μm.

Myospheres that were generated in the altered media began beating by day 8, while better maintaining their 3D architecture over extended periods of time (Figure 6A,B). The congruence of the aggregates for the heparin-containing media is shown by their attachment to the culture substrate, as demonstrated by their immunostaining for α7-integrin (Figure 6C)—whose expression is consistent with both explanted and in vivo cardiac tissue. Also consistent with a differentiated cardiac tissue is Cx43 expression (Figure 6D), as is the prominent staining of sMyHC throughout the tissue aggregates (Figure 6E). Moreover, the presence of the myocardial-specific transcription factor NKx2.5 within the aggregated tissue (Figure 7A–F) demonstrated the cardiac phenotype of the muscle tissue present in these cultures. Correspondingly, the lack of MyoD expression in these cultures (Figure 7G–I) suggests that contaminating skeletal muscle cells were absent from the cultures grown under the modified media conditions. The continued development of the aggregate cultures over time is demonstrated by continual contractile activity (Video S3) that is reminiscent of embryonic and adult heart tissue explants. Immunostaining for α-actinin at 4 weeks of incubation demonstrates the pervasive cardiac myosphere formation in these cultures (Figure 8A and Figure S1). The highly differentiated cardiac muscle that forms from the MDSCs is indicated by the organized sarcomeric patterning that is displayed within the aggregates, as shown by the pattern of α-actinin staining (Figure 8B–E). To further verify that contractility of the aggregates was due to the cardiac tissue formation and not twitching skeletal muscle cells, cadmium chloride was added to aggregate cultures. Within minutes, sphere contractile activity was completely abolished with the addition of cadmium chloride. This compound is a non-specific blocker of L-type Ca^++^ channels and Na channels [41]. Although skeletal muscle cells can sporadically twitch in culture [42], they do not respond to cadmium chloride treatment [43]. To date, these aggregate cultures have displayed a contractile phenotype that has persisted for at least 4 months (Video S4).

## 3. Discussion

Our laboratory has a long history of investigating signaling pathways and culture conditions necessary for promoting the cardiac differentiation of embryonic and adult cardiac progenitors and non-cardiac stem cell populations [31,44,45]. One example involved efforts to coax cardiac tissue formation from adult bone marrow stem cells, which required manipulations that affected histone methylation and has, to date, only generated partial cardiac phenotypes [32,36]. An alternative adult stem cell population that may contain a more intrinsic capacity to undergo cardiac differentiation are those associated with skeletal muscle. Many investigations have reported that muscle-derived stem cells may demonstrate cardiac potential under certain conditions, albeit with various degrees of success [23]. Both skeletal and cardiac muscles are derived from the early mesoderm and share some phenotypic similarities—most prominently, during their initial stages of differentiation [13,14,15,16,17]. Thus, skeletal muscle has long been thought to be an appealing candidate as a source of accessible stem cells whose differentiation could be redirected towards forming cardiac tissue.

A number of laboratories have reported achieving a contractile, cardiac phenotype with cells extracted from skeletal muscle. Some reported protocols require three-dimensional bioreactors [46], daily cell harvesting and replating [47], laborious hanging drop cultures [48] or co-culturing with murine cardiomyocytes [49]. The methodology used in this report did not require extensive tissue engineering protocols or co-culturing with purified cell populations. Rather, our protocol involved a less laborious, static culture system requiring simple cell passaging and feeding.

To examine whether stem cells associated with skeletal muscle show an intrinsic cardiac potential, we applied in vitro conditions used previously for cardiac progenitor cell and heart tissue explant cultures to our skeletal muscle cell preparation. The addition of dexamethasone was used to promote the cardiac cell potential and myosphere contractility [30]. We chose low-serum medium conditions that would be more selective for cardiac progenitor cells since the initial tissue preparation most likely contained multiple cell types. Indeed, initial cultures contained cell debris and very few phase-bright non-adherent cells. Cell proliferation became apparent by day 3 when small floating aggregates were visible under the microscope. These selective culture conditions did not promote the vigorous growth of other adherent cell populations, as few cells attached to the tissue culture plastic. Rather, the self-assembly of floating aggregates provided the selection of cells with the capacity to manifest a cardiogenic phenotype.

Our culture conditions did include factors that have been used in the development of early cardiomyocytes. Oxytocin has been shown to promote the development of cardiac myocytes from both adult and embryonic stem cells [38,39,50] and norepinephrine helps to maintain cardiomyocyte contraction [51]. Studies have demonstrated that the heart itself produces catecholamines via specialized intrinsic cardiac adrenergic (ICA) cells that arise in the embryo during the development of pacemaker and conduction tissue. These ICA cells remain in the heart and are present in the adult organ [52]. The addition of norepinephrine substituted for these ICA cells normally found in developing heart tissue.

Another agent that has been shown to be important for cardiomyocyte differentiation and function is reactive oxygen species (ROS). In mouse cells, the NADPH oxidases NOX1, NOX2, NOX3 and NOX4 are involved in generating ROS. In particular, with mouse embryonic stem cells, NOX4 or hydrogen peroxide (H_2_O_2_) promotes cardiomyocyte cell contraction [53]. The culture supplement B27 contains catalase, an antioxidant enzyme that breaks down H_2_O_2_. Thus, to increase ROS signaling, a low dose of H_2_O_2_ was added to the culture conditions. The contractility of myospheres was likewise supported by low amounts of TGFα. Studies have shown that epidermal growth factor receptor (EGFR) function is essential for maintaining heart contractility [54,55]. EGFR signaling is necessary for normal cardiac cell development [56], with both EGF and TGFα binding to the EGFR, also known as ErbB1 [57].

Contracting cells were first visible in floating aggregates by day 8, and their prevalence increased upon subsequent incubation times. Sensitivity to cadmium chloride demonstrated that these myospheres generated from MDSCs contained cells possessing L-type Ca^++^ channels similar to cardiomyocytes. The development of a cardiomyocyte phenotype was further supported by the expression of cardiac proteins. Specifically, we chose to analyze cells for proteins expressed during the development of the embryonic heart. Newly formed cardiomyocytes express NKx2.5, Cx43, sarcomeric α-actinin and sMyHC [58]. Early myocytes have also been shown to display markers associated with smooth muscle, including smooth muscle actin [59], SM22α [13] and calponin [15]. In addition, studies have demonstrated that NG2, a proteoglycan usually associated with pericytes, is expressed by embryonic cardiomyocytes [60]. Aggregates produced in our cultures also displayed Sca-1 and α-7 integrin, which are both associated with skeletal and cardiac lineage cells. Studies have demonstrated that precardiac cells and/or cardiomyocytes display Sca-1 [38,61] and α-7 integrin [62].

Our initial cell population isolated from skeletal muscle tissue was a heterogenous mixture of cell types. Antibodies were chosen to mark known stem and progenitor cell phenotypes: Sca-1 (satellite cells, fibroadipocytes), CD34 (satellite cells, fibroadipocytes, interstitial cells), CD90 (fibroadipocytes and CD133 cells), CD106 (muscle stem cells), NG2 (pericytes) and Pax3/7(satellite cells) [23,35]. The use of these markers to precisely identify specific cell populations harvested from adult skeletal muscle is limited, as many of these molecules are shared by multiple progenitor/stem cell types. Having said that, it is clear from our preliminary analysis, that the starting population that we harvested from the hindlimb muscle is unlikely to be skeletal satellite cells, based on the absence of CD34 and Pax3/7. More likely is that the harvested cells probably consist of a heterogenous mixture of other stem cell populations associated with skeletal muscle. It should also be noted that cell-type-specific markers are known to vary under in vitro cell culture conditions. Thus, investigators resort to functional assays, such as cell adherence, to purify cell populations [23]. Our protocol likewise incorporated the property of cell adherence into the selection of cells that may possess cardiogenic potential. Antibody binding was not used for cell selection. Subsequently, the immunocytochemical analysis allowed us to monitor changes in the aggregate to myosphere cultures with time, as the cells developed a cardiogenic phenotype, demonstrated by the expression of multiple cardiomyocyte-associated proteins and continuous cell contraction. Significantly, this contractile cardiogenic phenotype could be maintained for an extended period time with slight modifications to the culture conditions.

Both cardiac and skeletal muscles share an early mesoderm lineage that diverges as these phenotypes arise within the embryo [1,2,3,4,5,6]. This shared lineage appears to remain in the adult, as skeletal muscle stem cells manifest a cardiogenic potential under certain conditions. To determine whether these stem cells behave like CPCs, we established a simple, cell culture assay that yielded contracting cells displaying cardiac-associated markers. This in vitro system selected for the expansion of non-adherent cells that aggregated and displayed Sca-1-positive reactivity early during the culture period. Treatment in low-serum conditions and with additional cardiogenic agents allowed for the development of a cardiomyocyte phenotype from a cell population that should normally give rise to cell types found in skeletal muscle. Most importantly, this cardiac phenotype was stable, as cells continued to contract beyond four months in culture. Our results demonstrate that these selective cell culture conditions altered muscle stem cell maturation. Further analysis of the cell population selected and developed under these culture conditions will more fully elucidate the specific cardiomyocyte phenotypes produced in vitro and insights into the cellular pathways guiding the divergence of skeletal and cardiac muscle phenotypes. Moreover, the protocols described in this study would have utility for providing a source of easily obtained myocardial tissue for subsequent scientific investigations into cardiac function and biology.

## 4. Material and Methods

### 4.1. Stem Cell Isolation

All experimental procedures were approved the New York Medical College Institutional Animal Care and Use Committee. C57BL/6 mice from 3 to 8 weeks of age were euthanized with 3% isoflurane anesthesia prior to cervical dislocation [63]. Under sterile conditions, hindlimb muscles were harvested and cleaned of connective and adipose tissue. After the tissue was finely minced, the preparation was suspended in Iscove’s Modified Dulbecco’s Medium (IMDM: MilliporeSigma, Burlington, MA, USA) with 100 U/mL penicillin/100 µg/mL streptomycin (Pen/Strep; Corning, Glendale, AZ, USA), collagenase II (1 mg/mL; MilliporeSigma), dispase (2 U/mL; MilliporeSigma) and 2 mM calcium. Following a 1.5 h enzymatic digest at 37 °C with gentle mixing on an orbital shaker, the tissue slurry was passed multiple times through a 21-gauge needle to yield a cell suspension. These suspensions were diluted with 5× volume IMDM before being passed through a 40-gauge cell strainer and centrifuged at 500 g for 15 min. As a positive control for antibody reactivity, cell preparations from hindlimb muscle that contained satellite cells were isolated by extending the enzymatic digest for 2 h at 37 °C, with the resulting tissue slurry being dissociated by multiple passes through a 25-gauge needle. This rigorous treatment selected smaller-sized cells, including satellite cells [64]. Stem cells from adult mouse bone marrow were isolated and cultured as described previously [32,36]. For culturing and cell analysis, the resulting cell pellets were used in the experimental conditions listed below.

### 4.2. Skeletal Muscle Differentiation

Cells were suspended in IMDM containing 20% fetal bovine serum (FBS, MilliporeSigma) with Pen/Strep and plated in gelatin-coated 35 mm tissue culture dishes for six days. On day 7, the medium was replaced with IMDM containing 5% FBS, Pen/Strep plus 10 µg/mL insulin (MilliporeSigma). This low-serum medium was replenished every five days. Fully developed myotubes could be visualized within thirteen days of culture.

### 4.3. Analysis of the Initial Non-Adherent Cell Population

For immunocytochemical analysis of the initial non-adherent cell population within the skeletal muscle preparation, the resulting cell pellet was suspended in IMDM with Pen/Strep and 5% FBS. Following an overnight plating on tissue culture dishes, the non-adherent cell population was harvested and cytospun prior to staining. Antibodies were diluted according to the manufacturer’s specifications. Live cell staining was performed with the Sca-1, CD34, CD90 and CD106 antibodies (Invitrogen Thermo Fisher Scientific, Waltham, MA, USA). Formalin-fixed cells received the neural/glial antigen 2 (NG2; MilliporeSigma) antibody, as described below.

### 4.4. Cardiac Myosphere Differentiation

A modification of conditions reported for murine cardiosphere culture [65] was implemented for developing cardiac myospheres from MDSCs. Cells were suspended in IMDM containing Pen/Strep, 3.5% FBS, 2% B27 (Gibco Thermo Fisher Scientific, Waltham, MA, USA), 10 ng/mL epidermal growth factor (EGF; PeproTech; Cranbury, NJ), 5 ng/mL basic fibroblast growth factor (FGF; PeproTech), 5 U/mL heparin (MilliporeSigma), 10^−6^ M dexamethasone (MilliporeSigma), 25 µM hydrogen peroxide (H_2_O_2_; 30% stabilized) (Avantor, Radnor, PA, USA), 10^−7^ M oxytocin (MilliporeSigma) and norepinephrine (5 × 10^−6^ M; MilliporeSigma). The cells were cultured in tissue culture dishes for six days, with one change of medium at day 3. At day 6, floating spheres were collected and transferred to gelatin-coated dishes containing the medium with 5.0% FBS minus growth factors, B27 and oxytocin. Contractile activity of the myospheres was first visible by day 8.

### 4.5. Long-Term Myosphere Cultures

To maintain the contractile myosphere phenotype for an extended period, floating aggregates were collected on day 6 and cultured in gelatin-coated dishes containing IMDM with 5.0% FBS, Pen/Strep, 5 U/mL heparin (MilliporeSigma), 10^−6^ M dexamethasone (MilliporeSigma), 25 µM hydrogen peroxide (H_2_O_2_; 30% stabilized, Baker Chemicals), norepinephrine (5 × 10^−6^ M) and transforming growth factor alpha (TGFα; PeproTech) 0.5 ng/mL. Cultures were replenished with fresh medium every three days for the first two weeks and every six days thereafter.

### 4.6. Immunocytochemistry of Myospheres

For the immunocytochemical analysis of cellular spheres, floating aggregates were either transferred to collagen type I -coated chamber slides to promote loose cell adherence or embedded in collagen type I gels [66]. All antibodies were applied at the dilutions recommended by the manufacturers and used for immunostaining as described previously [31,44,63,67,68]. For Sca-1 and CD34 antibodies (Invitrogen Thermo Fisher Scientific), aggregates were stained live on ice for 30 min prior to neutral buffered formalin fixation. Staining with Pax3/7 antibody (Santa Cruz Biotechnology, Dallas, TX, USA) was performed on cytospun cells, permeabilized for 10 min with 1× PBS containing 0.3% Triton-X and 5% serum. For sarcomeric α-actinin, smooth muscle actin, (MilliporeSigma), calponin-1 (InnoGenex, San Ramon, CA, USA) and sarcomeric myosin heavy-chain (MF20; Developmental Studies Hybridoma Bank) antibodies, cells were fixed with cold methanol for 15 min prior to blocking [67]. For the detection with MyoD (BD Pharmingen, Franklin Lakes, NJ, USA) and Nkx2.5 (Invitrogen Thermo Fisher Scientific) antibodies, cells were formalin fixed for 30 min prior to a 1 h incubation with 1× PBS with 0.3% Triton-X, and 5% serum. SM22α (MilliporeSigma) antibody staining was performed on formalin-fixed spheres treated with 0.3% Triton-X plus 10% BSA for 30 min. Antibody to Alpha-7 integrin (Bioss Antibodies, Woburn MA, USA) was analyzed on formalin-fixed spheres treated with 0.3% Triton-X plus 10% BSA for 5 min. The NG2 antibody was applied to formalin-fixed aggregates that were blocked overnight at 4 °C with 1% BSA plus 5% serum. Connexin43 (Cx43; Invitrogen Thermo Fisher Scientific) was detected as described [44,63]. All fixed cultures were incubated overnight at 4 °C with primary antibodies. The secondary antibody concentrations used were those specified by the manufacturer (Jackson ImmunoLabs, West Grove, PA, USA). Cells were counterstained with 4′,6-diamidino-2-phenyindole (DAPI; MilliporeSigma) to mark the nuclei [63,68]. Cellular imaging was performed on a Zeiss LSM 710 or 980 laser scanning confocal microscope. Projection of Z-series image stacks acquired by confocal microscopy were produced using the ImageJ-based open-source Fiji software 2.16.0 (https://fiji.sc). and Helicon Focus 8.2.5 (Helicon Soft Ltd., Kharkiv, Ukraine).

### 4.7. Cell Counts and Determination of Cellular Yields in Long-Term Cultures

The yield of cells that aggregated into cardiac myospheres was determined by morphometric analysis of long-term cultures that developed from the stem cell populations harvested from skeletal muscle. The densities of cells contained within and outside of the myospheres were discerned by protocols described previously [63]. DAPI-stained cultures were imaged using a Zeiss LSM 980 laser scanning confocal microscope and manually counted for nuclei with Fiji image processing software 2.16.0. To determine cellular yields within the cultures, nuclei were counted within multiple fields for each culture. Areas of each field that consisted of the myospheres and the regions surrounding these aggregates were computed from microscopic images into pixel densities using Adobe Photoshop. The number of cells within the myospheres, as well as the non-muscle cells in the surrounding areas, were calculated from the number of nuclei counted as a function of the overall ratio of the pixel areas of these respective areas within the cultures. The total yields of myosphere cells within the cultures were determined based on the ratio of the area counted compared to the total area of the sections.

### 4.8. Contractility and Live Imaging Analysis of Cardiac Myospheres

Contractile myosphere cultures were examined daily by visual inspection using a Zeiss AxioVert culture microscope. Still images and videos of live cultures were acquired on a Zeiss Axio Observer inverted microscope and a Zeiss AxioVert culture microscope. The development of cardiac cell contractility from MDSCs was verified by the addition of 0.5 mM cadmium chloride (MilliporeSigma) to beating aggregates [46,69].

## Data Availability

The data supporting this study are contained within the article and Supplementary Materials.

## Supplementary Materials

The following supporting information can be downloaded at: https://www.mdpi.com/article/10.3390/ijms26062683/s1.
