# Supplementary material for: Stem Cells Associated with Adult Skeletal Muscle Can Form Beating Cardiac Tissue In Vitro in Response to Media Containing Heparin, Dexamethasone, Growth Factors and Hydrogen Peroxide
Source: Int J Mol Sci. 2025 Mar 17;26(6):2683. doi: 10.3390/ijms26062683 (PMC11942180; doi:10.3390/ijms26062683)
Supplement: Supplementary file 1 [file ijms-26-02683-s001.zip › Supplemental Figure Legends.pdf]

Supplemental Figure Legends

Video S1. Beating aggregate at 10 days of incubation under the initial media conditions.

Video S2. Beating tissue at 28 days of incubation under the initial media conditions.

Video S3. Beating myosphere at 28 days of incubation under the modified media conditions.

Figure S1. Panoramic view of Day 28 myosphere cultures. Panoramic slice from a 35 mm dish showing a portion of a day 28 culture containing MDSC-derived contractile myospheres, that was imaged following fluorescent-labeling with α-actinin-specific antibody. This image shows an individual example of the modified culture protocol described in this manuscript, which to date has been repeated 14 times. These cultures begin with an initial plating of 2×10^6 MDSCs, most of which either do not survive the initial culture or became attached to the dish. On day 6, floating aggregates are transferred to a fresh 35 mm dish. Cell counting indicated that by day 14, total cells remaining in the cultures after dissociation = 1.36×10^5/dish (std. dev. 1.12×10^4; n = 3). By day 28 the number of myosphere cells/dish = 6.1×10^5 (std. dev. 1.2×10^4). Scale bar = 200 μm.

Video S4. Beating myosphere at 4 months of incubation under the modified media conditions.
