# Supplementary figures and images for: Stem Cells Associated with Adult Skeletal Muscle Can Form Beating Cardiac Tissue In Vitro in Response to Media Containing Heparin, Dexamethasone, Growth Factors and Hydrogen Peroxide
Source: Int J Mol Sci. 2025 Mar 17;26(6):2683. doi: 10.3390/ijms26062683 (PMC11942180; doi:10.3390/ijms26062683)

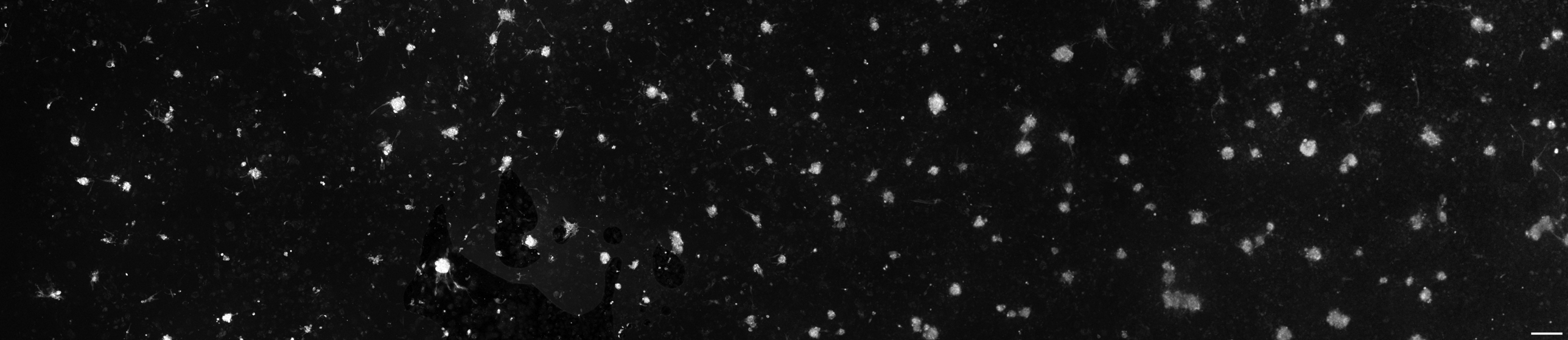

Supplement: Supplementary file 1 [file ijms-26-02683-s001.zip › Figure S1 - Panoramic view of Day 28 myosphere cultures.tiff]
